# Alternative Molecular-Based Diagnostic Methods of Plant Pathogenic Fungi Affecting Berry Crops—A Review

**DOI:** 10.3390/molecules24071200

**Published:** 2019-03-27

**Authors:** Dominika Malarczyk, Jacek Panek, Magdalena Frąc

**Affiliations:** Institute of Agrophysics, Polish Academy of Sciences, 20-290 Lublin, Poland; d.malarczyk@ipan.lublin.pl (D.M.); m.frac@ipan.lublin.pl (M.F.)

**Keywords:** *Colletotrichum acutatum*, *Verticillium* spp., *Phytophthora* spp., *Botrytis cinerea*, PCR, qPCR, molecular identification, phytopathogenic fungi, strawberry, organic agriculture

## Abstract

Increasing consumer awareness of potentially harmful pesticides used in conventional agriculture has prompted organic farming to become notably more prevalent in recent decades. Central European countries are some of the most important producers of blueberries, raspberries and strawberries in the world and organic cultivation methods for these fruits have a significant market share. Fungal pathogens are considered to be the most significant threat to organic crops of berries, causing serious economic losses and reducing yields. In order to ameliorate the harmful effects of pathogenic fungi on cultivations, the application of rapid and effective identification methods is essential. At present, various molecular methods are applied for fungal species recognition, such as PCR, qPCR, LAMP and NGS.

## 1. Introduction

Organic fruit production has been increasing constantly in recent decades and has also increased its market share in the production of food worldwide. Strawberry, blueberry and raspberry fruits are important products of Central Europe and increasing consumer demand to introduce organic methods of fruit cultivation is a major reason to seek alternative ways to reduce losses. The main concerns of food producers are diseases caused by fungi, these pathogens attack plants and fruits from the early stages of sowing to the moment of market sale, thereby causing the unpredictable spoilage of products. The crucial plant pathogens discussed in this review are those from the genera *Verticillium* and *Phytophthora* as well as species such as *Colletotrichum acutatum* and *Botrytis cinerea*.

For many years, morphological methods of identification have been applied for the purposes of recognizing the causal agents of soil-borne diseases. However, these traditional methods are time consuming, error-prone and occasionally inaccurate. Because of these disadvantages, more efficient methods, such as adopting analytical techniques which function at the molecular level are being used more frequently. Polymerase chain reaction (PCR) based methods allow for the multiplication of targeted fragments of DNA over a short periods of time in order to obtain enough genetic material for further research. The purpose of this review is to gather together the most important information concerning the molecular methods of identifying important berry pathogens.

## 2. Organic Plantations and Fungal Pathogens

The area of land under organic cultivation worldwide has increased fivefold since 1999. Since then, this area has increased from 11 to nearly 58 billion hectares in 2016, and the area has increased in every continent. As of 2016, Central Europe had more than a billion hectares increase in organic arable land area in comparison to 2007 which is [1]. Almost one-quarter of the organic lands on the globe [1]. Europe has nearly half of the worlds harvesting area of strawberry, blueberry and raspberry fruit [2] and Central European countries produce big share of those fruits with 335,000, 113,500 and 30,000 tones berries produced in 2017, respectively. What is more, this region holds quarter of the world’s raspberry harvesting area [2]. Europe noted 320% increase in production of strawberries, blueberries and raspberries from 2007 to 2016 [2]. Eleven percent of worlds strawberry farmlands in 2016 was organic and what is more, in European Union, nearly 20% of berries were grown in organic agricultures [3]. In addition to an increase in the land area of organic farming activity in Europe, the sales market for organic farm produce is also growing significantly. The enlargement of the market in the years 2000–2015 was more than 300% in both areas. The main reason for the higher sales indicator from 2005 to 2014, was the constant increase in the consumption of ecological foods [4]. Poland and Hungary, as two Central European countries, have relatively small markets. Yet, they produce a large share of the organic crops in the free trade area and that makes them important exporters of ecological products [5]. Poland alone was the 3rd biggest exporter of prepared fruits in the world with 429,600 tonnes of fruits exported in 2015. Strawberries and fruit juices are also important export products with 16,500 and 79,000 tonnes, respectively, being exported in 2015. In 2016, Poland was also the 3rd biggest fruit exporter in the world [2].

Fields cultivated using organic methods are particularly exposed to pathogens due to the exclusion of chemical spraying for the purposes of disease management. Central Europe countries have a relatively warm and humid climate [6], which are ideal conditions for the development of fungal diseases. Berries are especially vulnerable to the harmful effects of fungal pathogens due to their thin cell walls, growth close to the wet soil surface and exposure to rainfall. Fungal diseases can lower the yields even down to 50%, even with application of appropriate chemical sprayings [7,8]. The optimal treatment is even more difficult due to the fact that diseases can remain dormant even for many years, waiting for optimal conditions to attack theirs hosts [9,10,11,12]. The pathogens which repeatedly attack cultivations of soft fruits, as well the fruit harvest in cold storage, are typically various species of fungi. The most common and threatening fungi in Central Europe are those of the *Verticillium* and *Phytophthora* genera, as well as *Botrytis cinerea* and *Colletotrichum acutatum* that are involved in yield and quality losses of soft berry fruits. The abandonment of conventional fungicides creates the need for early and effective detection methods of causal agents of plant diseases to prevent the spread of disease to the entire crop during current and future growing seasons.

## 3. Fungal Pathogens—Characteristics, Occurrence, Properties and Threats to Organic Farming

### 3.1. Verticillium *spp.*

**Figure 1:**
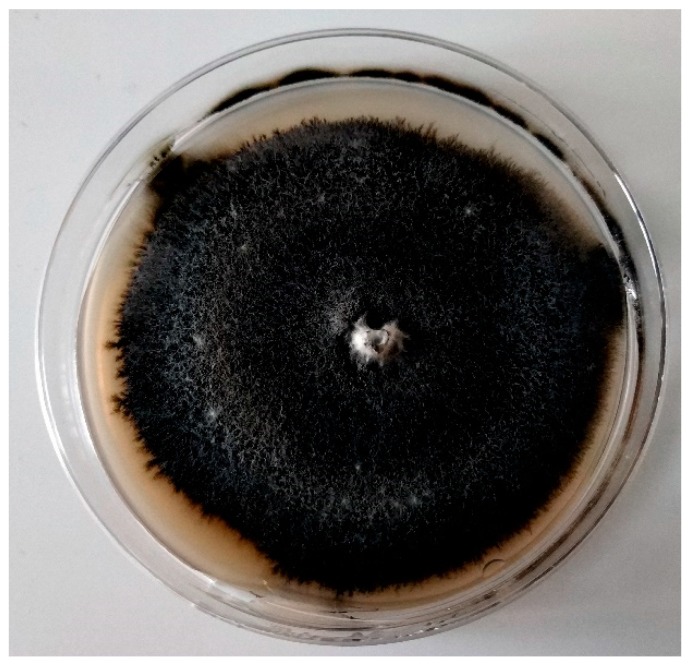
*Verticillium* sp. isolated on PDA medium from ecological strawberry plantation after 10 days of incubation in 22 °C.

Fungi belonging to *Verticillium* spp. attack various species of fruits, vegetables, flowers and forest trees, including many species of soft fruits. Theirs host range includes: strawberry (*Fragaria × ananassa* Duchesne*),* red raspberry *(Rubus idaeus),* black raspberry *(Rubus occidentalis)*, thimbleberry *(Rubus parviflorus)* and some cultivars of blackberry *(Rubus ursinus)*. Only 5 families of plants, such as: *Cactaceae, Gramineae, Gymnospermae, Monocotyledoneae* and *Polypodiaceae*; are reported to be resistant or immune to soil-borne disease called Verticillium wilt [13,14,15]. *V. dahliae* and *V. albo-atrum* are two species with the most significant pathogenicity amongst the 10 which have been distinguished recently: *V. albo-atrum, V. alfalfa, V. dahliae, V. isaacii, V. klebahnii, V. longisporum, V. nonalfalfae, V. nubilum, V. tricorpus* and *V. zaregamsianum* [16]. Most of *Verticillium* species are not host-specific, and symptoms of infection vary between carriers, thus there are no universal signs of the disease on the plant. Some of the species of the genus may easily be distinguished by the shape of their microsclerotia and the length of the conidia they form on hosts and potato dextrose agar (PDA) [16]. Hyaline colonies formed on agar plates are whitish, turning darker with time (Figure 1), and they produce bountiful conidia [17,18,19,20]. The fungus degrades the cell walls of the host with several enzymes, which causes necrosis and other symptoms [21]. One of these enzymes is polygalacturonase [22] and its production level is related to the degree of fungus pathogenicity [23]. Moreover, *Verticillium* wilts are easily spread via contaminated plant material, soil and equipment. The conidia-producing specimen—*V. albo-atrum* is spread via air currents. When the wilt is securely situated in soil, it can survive for more than 25 years [9,15]. As a result of infection, the probability of the infected plant producing fruit is vastly reduced [15]. The plant often becomes infected through wounds in the roots [24]. The most effective way to control the disease is through the elimination of contaminated plants from the field [21], thus the rapid and efficient identification of the pathogen is obligatory.

### 3.2. Phytophthora *spp.*

**Figure 2:**
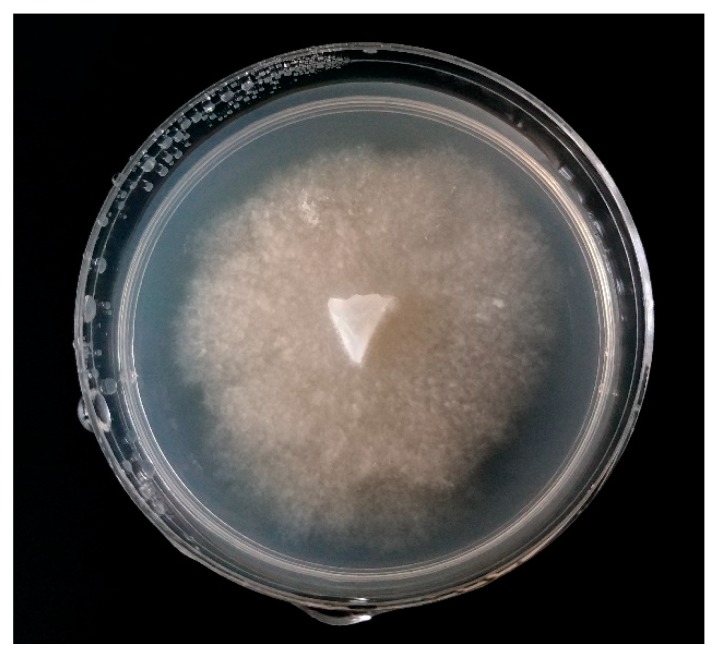
*Phytophthora* sp. isolated on PDA medium from ecological strawberry plantation after 10 days of incubation in 22 °C.

The *Phytophthora* genus includes at least 124 described species [25]. The pathogen infects a wide variety of plants worldwide, and its introduction to a new continent can damage the whole ecosystem. In Victoria, Australia, *Phytophthora cinnamomi*, as well as 13 other species in the taxa is a leading pathogen which has been discovered in the soil. The fungus is an important threat to native plants on the continent and can harm fruit plantations [26]. European and American strawberry and raspberry plantations are also attacked by *Phytophthora*, causing crown and leather rot, resulting in the dieback of plants and severe harvest reduction. Fungi occurring in the soil, belonging to the *Phytophthora* spp., are not host specific, and are a threat for both, strawberry and red raspberry. Disease manifestations on fruits are similar to those caused by *Colletotrichum acutatum* or *Verticillium dahliae* and are often misdiagnosed. In a study from 2018, Wilcox’s team proved that the main raspberry pathogen present in soil was *Phytophthora rubi* and that it was the main causative late-summer symptom of disease [27,28,29,30]. The selective media utilized for *Phytophthora* sp. isolation are V8 juice agar (V8) and cornmeal agar (CMA) with the addition of various antibiotics. Antibiotics and specific antifungal agents are added to inhibit the development of bacteria and other than *Phytophthora* sp. fungi competing for resources. The morphological identification of colonies may be difficult because random mutations and the different growth conditions present in nature may lead to a variability in the phenotype of the species. An overlap of morphological features of the genus also impedes accurate identification. Nevertheless, the size of the sporangium and papilla, as well as the appearance of sporangia are commonly considered for the classification of the fungus (Figure 2) [31].

### 3.3. Botrytis cinerea

**Figure 3:**
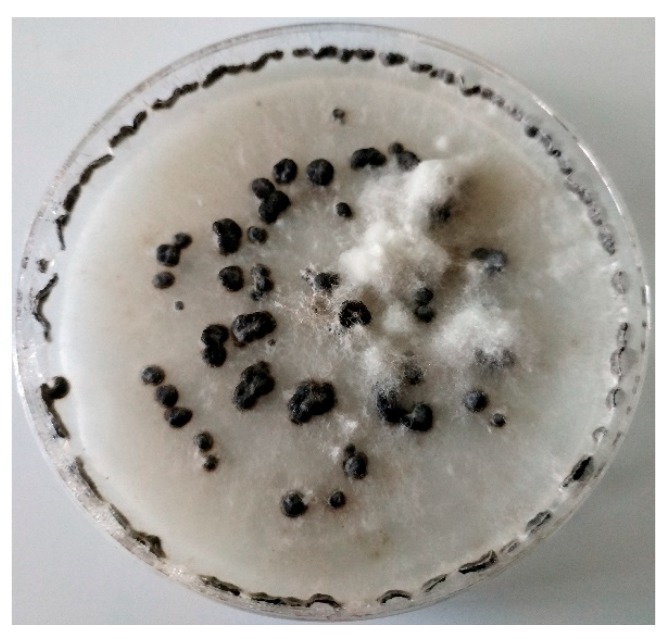
*Botrytis cinerea* isolated on PDA medium from ecological strawberry plantation after 10 days of incubation in 22 °C.

*Botrytis cinerea,* which causes gray mold, is an important necrotrophic fungus infecting more than five hundred species of plants [32,33,34,35], including strawberry and raspberry [10,34,36,37]. When considering the impact of fungus on fruit production, it took second place in the list of top ten fungal pathogens of molecular plant biology in 2012 [38]. The susceptibility of strawberry plants to the fungus is known to severely decrease harvests, even down to 50% [36]. The presence of the pathogen may remain hidden. In that case necrotrophic disease may be triggered by outside conditions, such as rainfall, a relative humidity higher than 80% for at least 4 hours, and an appropriate ambient temperature of 2–28 °C [10,11,35,39]. The pathogen can propagate on harvested strawberry, raspberry, blueberry and blackberry fruits at temperatures above freezing, which is a significant problem for the cold storage of soft fruits [40]. The disease may occur on fruits at any time from seedling to sale, what makes it difficult to predict and effectively counteract [38,41]. However, it is known that ripe fruits are most susceptible to infection [42]. In order to infect the host, spores are produced and spread, mainly conidia distributed by wind, rain and insects [43,44]. Fungus germ tubes and appresorias may produce an extracellular matrix, which helps them to attach to the cell walls of hosts and degrades them with enzymes [45]. In some cases, pathogens may penetrate the cuticle without the secretion of enzymes [46]. Invasions through wounds and blossoms are also often detected [40,47]. Pollinating insects such as honey bees have the potential to disperse disease in a similar fashion [48]. *B. cinerea* colonies grown for 7 days at room temperature on a PDA medium produces abundant whitish mycelium, which becomes darker with time (Figure 3). However, some of the isolates may have diminutive mycelium and produce a yellow pigment on PDA, which is undeveloped on other commonly used medias [49,50]. Conidia, ovoid or ellipsoid and one-celled, are on average 8–13 µm in length and 4–7 µm in width and are dispersed by the air [50].

### 3.4. Colletotrichum acutatum

**Figure 4:**
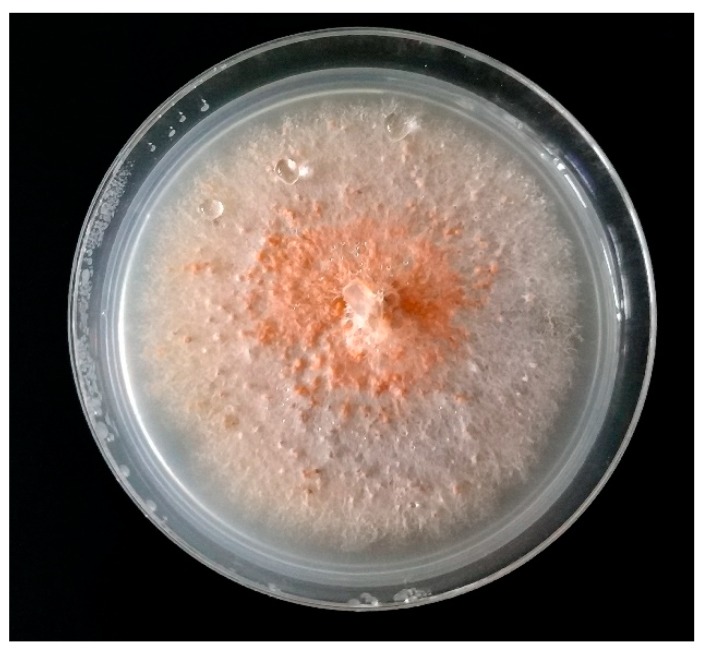
*Colletotrichum acutatum* isolated on PDA medium from ecological strawberry plantation after 10 days of incubation in 22 °C.

Anthracnose is a disease caused by *Colletotrichum acutatum.* The fungus attacks a wide range of plant species around the world [51] and is known mainly as a pathogen of strawberries. *Colletotrichum* spp. have been evaluated as the 8th most important fungal pathogen in plant biology [38]. Infection may remain dormant until the fruit is stored, and then cause losses of up to 100% [37]. The fungus is necrotrophic lifestyle, and causes black spots to form on strawberry fruits, additionally attacking roots, crowns and leaves [52,53,54,55]. The colonies of the pathogen isolated on PDA are whitish at first, becoming gray with time and the reverse of the Petri dish is pink or pale orange (Figure 4). Conidia, observed under a light microscope, are 8–16 × 2.5–4 µm in size, one-celled, straight, but pointed at the end (fusiform). Conidial appresoria are grey and globular in shape [54,56]. The fungus is mainly dispersed by rain, and can enter the host via any plant tissue. Dispersal of conidia can reach as far as 1.75 m through splashing and the infection of one plant in the field by the pathogen proceeds to the whole cultivation [57]. Most frequently *C. acutatum* infects strawberries through the crown, as there is a humid microclimate [58]. The fungus is capable of wintering in the soil for at least two winters with temperatures falling below 0 °C, this causes anthracnose to develop in subsequent years [12,59]. This is the reason why optimal treatment for the disease is necessary not only for the harvest in the current year, but also for consecutive seasons.

## 4. Detection Methods of Plant Pathogenic Fungal Species

### 4.1. Traditional Methods

Traditional methods of fungal pathogen identification include experienced scientists studying their morphological attributes such as colony appearance and the production of asexual structures on microbiological media or on the host. Samples isolated on adequate agar media may be observed using a light microscope to track the presence of the slightest structures. This method is time consuming and only mature colonies may be evaluated. Occasionally colonies have to meet certain conditions to produce conidia and this may cause inconvenience in laboratory work-flow [60,61,62]. Selective medias have been proposed and used for identification, for example Botrytis Selective Media (BSM) for *Botrytis cinerea* [63]. The recognition of external infection symptoms induced by fungi on theirs hosts can also be used to verify the pathogen, although most species are not host specific and plants may be inhabited by many fungi. The lack of carrier specificity and symptom differences between plant populations at different latitudes makes an accurate identification based only on the morphology of the colonies very difficult or even impossible. Furthermore, interpretations of the pathogen’s morphology are subjective and highly reliant on one’s experience. The human factor may lead to an incorrect identification of the pathogen, causing misguided plant protection activities.

### 4.2. Molecular Methods

In recent years, molecular methods are being more and more willingly used by researches in many fields. They are also widely applied in order to identify fungal diseases or for recognition of new fungal species and the description of pathogen populations. The identification of fungi is in fact more accurate when molecular markers are applied, compared with assignment to the species based only on morphology [64,65] and the technique may be used by personnel without specific taxonomic expertise [66].

The polymerase chain reaction (PCR) process includes the in-vitro amplification of targeted genes from previously isolated DNA [67]. After the reaction, an electrophoresis is performed on the agarose gel of the fragments produced which are stained with EtBr (Ethidium bromide) or SYBR Green. The occurrence of a fragment of specific length confirms the presence of a pathogen. Further sequencing of the products may also be performed to ensure the specificity of the obtained amplicon.

The modification of the method, allowing the observation of the amplification results in real-time and the quantification of the genetic material in the sample is quantitative PCR (qPCR) [68,69]. An assay has many advantages in comparison with PCR. The reaction does not require further electrophoresis, as the analytical techniques used in the reaction allows for the observation of the size of the fluorescent signal which is proportional to the amount of amplified DNA. The qPCR technique also allows for the analysis of from 96 to 386 samples simultaneously as it is performed on plates [66]. A comparison of PCR with qPCR by Garrido’s team demonstrated that the qPCR reaction is 100 times more sensitive compared with PCR when it is applied to the identification of plant pathogenic fungi on strawberry fruit [70]. 

Loop-mediated isothermal amplification (LAMP) is another method utilizing DNA polymerase, with the distinction of a constant temperature throughout the whole reaction and the utilization of two or three sets of primers. The assay is highly specific due to the presence of a larger number of primers in comparison with PCR. For the same reason, the reaction is insensitive to contamination with non-specific DNA [71]. LAMP can be verified directly through the examination of a color change in the samples or by electrophoresis [66]. What is more, *Bst* polymerase which are often used in the reaction are less susceptible to inhibitors compared with *Taq* polymerase. Therefore, LAMP does not always require DNA isolation and may be performed directly from the environmental sample [72]. Also, due to the constant temperature character of the reaction LAMP doesn’t require specialist equipment such as a thermocycler [73]. 

Next-generation sequencing (NGS) belongs to the methods that were developed after automated Sanger assays—‘first generation’ for sequencing genetic material. The most important advantage of NGS is the ability to sequence billions of nucleotides during one run, thus sequencing whole genomes has become available for academic uses [74,75]. Table 1 presents the data concerning genome assemblies of fungal pathogens described in this paper. Although most of the genomes have already been deposited in the international bioinformatics database, the information is not sufficient to describe all features and functions of these organisms and still there are a lot of work to get to know them well.

It is known that inter- and intra-specific variability are likely widespread in fungi. This fact has implications for research on fungal taxonomy, phylogenetics, evolution, and population genetics. However, described methods can be successfully used to portray intra- and inter-specific variability of microorganisms occurring in the environment, in particular pathogenic fungi, but also the other fungi. More specific approaches have been already developed, such as: Restriction Fragments Length Polymorphism (RFLP), Random Amplification of Polymorphic DNA (RAPD), Terminal Restriction Fragments Length Polymorphism (tRFLP) and Amplified Fragment Length Polymorphism (AFLP). When planning the experiment, it is important to remember to take into account native strains of fungi that are present in the habitat, as intra-specific variability can affect the analysis [76,77,78]. It is also important to highlight that detection limits of the fungi reported in this work can only be treated as guidelines and not certainty, as those limits are dependent on numerous factors, including the type of medium, age of the culture or isolation methods.

#### 4.2.1. *Verticillium* spp.

Many studies have utilized a comparison with the *ITS* region in the phylogenic analysis of *Verticillium* spp. [19,79]. Some of protein-coding genes were also used for distinguishing species, such as: cytochrome c oxidase III (*COX3*), NADH dehydrogenase subunit I (*NAD1*)*,* actin (*ACT*), elongation factor 1-α (*EF*), glyceraldehyde-3-phosphate dehydrogenase (*GPD*) and tryptophan synthase (*TS*) [16,19]. The detection of *V. dahliae, V. tricorpus* and *V. albo-atrum* in strawberry fields was performed using five simplex loci, including *ACT*, *EF*, *GPD*, *TS genes* and *ITS* region. The discrimination between the species was performed using multiplex PCR with listed markers with an irrefutable outcome [79]. The *ITS* region sequencing was again successfully used for the confirmation of *V. dahliae* as an olive tree pathogen [20]. However, Yu’s team was not able to distinguish between *V. dahliae* and *V. longisporum* based only on the analysis of the *ITS1*-5.8S marker, thus *COX3* and *NAD1* genes were also included in the study to make the analysis more specific [19].

Lievens’ team developed a real-time PCR assay for the identification and quantification of 3 species linked to *Verticillium* wilt on tomato plants. The targeted marker was the *ITS1* region. Primers were specific to all three targeted species, those being *V. albo-atrum, V. dahliae* and *V. tricorpus*, and the amplification did not occur with any of the additionally tested fungi [80]. The marker gene was also used in a similar study to estimate the number of strawberry pathogens in the soil samples, including *Verticillium* spp. The method was able to detect 17.7 pg of the *V. dahliae* DNA [81]. The *ITS* marker was also applied in the quantification of *V. dahliae* in affected strawberry roots and soil. The detection limit for the fungus genetic material was 0.93/µL pg and the lowest amount of *V. dahliae* detected in soil equaled 10.48 pg/µL [82]. A different study, demonstrating differentiation between *V. dahliae* and *V. longisporum* and the identification of *V. tricorpus* by qPCR was published in 2011. The amplification of the *ITS* region of *V. tricorpus* was performed with specific primers and was able to detect 0.1 microsclerotia/g of soil. *V. dahliae* and *V. longisporum* were distinguished based on the sequence of the *β-tubulin* gene, and the assay was able to track as little as 0.5 fungus microsclerotia/g of soil [83,84]. An analysis of the abovementioned *β-tubulin* primers, with the addition of an *ITS* marker, were also used for the identification of *V. longisporum* oilseed rape in qPCR. The *β-tubulin* primers were specific for the targeted specimen, however they did not detect 3 of the isolates. This may confirm that the new taxonomy of fungi proposed by Inderbitzin is correct [16]. The *ITS* marker was also highly specific to the genus, and detected 0.56 fg of fungal DNA. Despite that, the marker was not able to distinguish *V. longisporum* from other species in the *Verticillium* genus used in the study. Another disadvantage of the *ITS* primers was that they were also specific for *B. cinerea* and a few *Alternaria* isolates [85]. The quantification of *V. dahliae* in lettuce leaves was successfully performed by Klosterman. The assay amplified the *β-tubulin* targeted gene and was able to detect 2.5 fg of fungal DNA 21 days after the inoculation of the pathogen on the plant [86]. Another marker successfully used in qPCR was an intergenic spacer of genomic DNA (*IGS*). The reaction was performed to identify *V. dahliae* and *V. tricorpus* pathogens on various plants, including strawberry. Two pairs of created primers were specific only to *V. dahliae* and *V. tricorpus*, and none of the non-targeted species were amplified. Bilodeau’s team succeeded in detecting as little as 3 fg of fungus DNA with the assay. Also, they estimated the number of copies of *IGS* in the pathogen genome, comparing the amplification of the aforenamed region with the single-copy genes such as: endochitinase, *β-tubulin* and glyceraldehyde-3-phosphate dehydrogenase (*G3PD*). They averaged the number of the *IGS* region in different isolates to 46 copies in the haploid genome with the qPCR assay. In the study, an additional specific primer pair of *IGS* for *V. albo-atrum* was designed, however, in an initial examination only *V. dahliae* was apparent in the samples. This is why only the first pair of primers was used in further stages of research [87]. Another gene targeted for the detection of *V. dahliae* in potato crops was an extracellular trypsin protease (*VTP1*). The PCR technique detected 25 pg of fungal DNA, but primers were also specific for *V. longisporum*. The qPCR technique was 10 times more sensitive than the PCR technique with the same primers. Also, the duplex qPCR technique additionally targeting the potato actin gene was developed and was able to detect as little as 0.25 pg of *V. dahliae* DNA [88]. The multiplex approach was further investigated with the *VTP1* gene of *V. dahliae* and the internal control actin gene (*ACT*) of *Solanum tuberosum.* The assay was performed in field conditions with remarkable reliability [89]. The quantification of soil-borne diseases on strawberry fruit was performed with the application of *ITS1* primers as described previously by Lieven’s team [80,81]. In agreement with their discoveries, in the Ozyilmaz study, the marker was specific for at least 5 of the *Verticillium* species. The reaction detected 0.6 pg of pathogen DNA [81]. The identification of *Verticillium* species in soil was also performed by the Tzelepis’ team using a qPCR assay with newly designed primers for *V. dahliae, V. longisporum, V. tricorpus* and *V. albo-atrum*. The detection level equaled 5 and 6 fg DNA/g of soil for *V. longisporum* and *V. dahliae*, respectively, and the last two fungi were not detected in the soil samples [90]. The most important information described above are summarized in Table 2.

A LAMP assay with newly designed primers for the selection of previously established random amplified polymorphic DNA (RAPD) makers was performed by Moradi’s team. The reaction was able to detect as little as 50 fg DNA from *V. dahliae* isolates, which was 10,000 times more sensitive than that conducted by the team nested-PCR. What is more, none of non-targeted species were amplified in the reaction, including other soil-borne pathogens and other *Verticillium* species [95].

The phylogenic analysis of *Verticillium dahliae* with the application of NGS was completed in 2013. The team also acquired a draft genome sequence of the fungus [96]. As a continuation of the study, Faino’s team assembled a complete and gapless genome of the pathogen [75,97]. Further genome sequencing of *V. dahliae* genetic material obtained from strawberry pathogenic strands resulted from *c.* 33 Mb assembly with 44–80-fold coverage [98]. With the *de novo* genome sequencing of *V. nonalfalfae*, Jelen’s team additionally identified the mitochondrial genome of the fungus. The size of the acquired mitochondrial sequence averaged 27% GC content and close to 26 kb of nucleotides [99]. The complete *V. longisporum* genome assembly acquired in 2018 was estimated at 70 Mb, and the mitochondrial genome equaled *c.* 27 kb [100].

#### 4.2.2. *Phytophthora* spp. 

In order to identify species in the *Phytophthora* genus, *ITS1* and *ITS2* were amplified using PCR and sequenced. The variability of *ITS2* was less significant in comparison with *ITS1*, but both of the markers were useful in species identification within the genus [101]. Ristaino’s team developed an assay for rapid identification within the genus. They amplified the *ITS* region, and for species recognition, employed restrictions enzymes. Additionally, they developed a specific primer for *P. capsici* (*PCAP*) [102]. Cooke’s team analyzed the *ITS* marker of various *Phytophthora* species, containing red raspberry and strawberry pathogens for phylogenic purposes [103]. Also, they designed genus-specific primers for the *Phytophthora* spp. with the application of the cytochrome oxidase I (*COX1*) gene with high specificity. In order to establish a greater distinction between *P. ramorum, P. nemorosa,* and *P. pseudosyringae*, they used nested PCR with species-specific primers. The primers amplified targeted species sufficiently and did not produce a sequence with any of the non-targeted species added in the study [104]. The *ITS* marker was also used for the identification of the *Phytophthora* pathogen causing rot of cranberry [105]. Further, the multilocus approach for the phylogenic analysis of the genus was developed by Blair’s team. In the study, 27 loci were targeted, and 7 loci were successfully amplified: 28S ribosomal DNA, 60S ribosomal protein L10, *β-tubulin*, elongation factor 1 α, enolase, heat shock protein 90 and the *TigA* gene fusion protein. The first two markers were both amplified within the genus, but the second one was not long enough (496 bp) to deliver sufficient phylogenetic information. *β-tubulin*, enolase and *TigA* loci provided satisfactory phylogenetic information among the *Phytophthora* genus. Next, heat shock protein 90 and elongation factor 1 α produced a moderate level of information among most clades [106]. In a continuation of the cited study, Martin’s team provided an additional analysis of 4 mitochondrial loci within the genus. The markers used in the report: cytochrome c oxidase subunit II (*COX2*), NADH dehydrogenase subunit IX (*NAD9*), 40S ribosomal protein S10 (*RPS10*) and protein translocase subunit SecY (*SECY*) loci, and the phylogenic tree was comparable with the one constructed in the former study [25,106]. In a paper published in 2014, an analysis of the *ITS* marker was once more applied to the identification of *Phytophthora* spp. obtained from nursery plants, irrigation water, and potting media. Sixteen species within the genus were identified, then isolates from *P. citricola* complex were additionally sequenced with *β-tubulin* primers to ensure the specificity of the obtained products [107].

The first employment of the qPCR assay for monitoring *Phytophthora* spp. in different host tissues showed that the method may in fact be successfully used for this purpose. The assay contained the design of new primers for *P. infestans* and *P. citricola*: specific GC rich nuclear satellite DNA with unknown function, and *ITS1*, respectively. The reaction was able to detect 1 µg of *P. infestans* and 10 ng of *P. citricola* through template DNA in the sample [108]. The detection of *Phytophthora* spp., as well as the species-specific identification of *P. ramorum* was also further performed. Both pairs of primers were targeted for the *ITS* gene. Also, primers for the detection of false-negatives were used with the implementation of the *COX* gene. The genus-specific primers amplified all of the *Phytophthora* species in the study, however, non-targeted isolates of *Pythium* were also amplified [109]. *P. cactorum* was one of the targeted species in the qPCR assay used for the identification of strawberry pathogens in the soil. The fungus was successfully detected in the amount of 8.6 fg/μL through the amplification of the *ITS* region with specific primers [81]. The *ITS* marker was also used for the identification of strawberry soil-borne pathogens. The qPCR assay detected 1 pg of *P. cactorum*’s DNA per 1 g of soil [110]. The identification of the pathogen causing late-summer disease symptoms on raspberry fruit was performed by Weiland’s team with the application of qPCR. Even though the disease was first connected to the presence of *Verticillium dahliae*, diagnostic tests produced conflicting results. Ultimately, qPCR indicated that the main cause of the late-summer symptoms of disease was *Phytophthora rubi* [29]. The multilocus approach was performed for the identification of *P. colocasiae* with the application of 3 markers: RAS-related protein (*YPT1*), G protein alpha-subunit (*GPA1*) and phosphoribosylanthranilate isomerase (*TRP1*) genes. All of the amplifications were successful, thus the best sensitivity was demonstrated by *YPT1*, with the detection of 12.5 fg of fungal DNA. The pathogen was amplified using a qPCR assay 15 hours after the artificial infection of the plant; 3 hours earlier than in PCR [111]. The simultaneous detection of two pathogens, *P. nicotianae* and *P. cactorum* from strawberry tissues in the qPCR assay was also performed. The primers designed for the *ITS* region and the *YPT1* gene were utilized with sufficient results. The assay was able to detect 10 fg and 1 pg of targeted DNA from *P. nicotianae* and *P. cactorum*, respectively [112]. The triple approach of detecting *Malus Miller* pathogens using qPCR was also verified in a recent study. Three pairs of primers for enolase (*ENOL*), ras-like protein *YPT1* and *HSP90* gene sequences were designed for *P. hibernalis*, *P. cambivora* and *P. syringae*. The primers were capable of simultaneously detecting 20 pg of the two first species and 0.2 pg of the third fungus genomic DNA [113]. Table 3 summarizes selected facts containing the primer sequences used in the above-described papers.

The *ITS* marker in the LAMP assay was used for the detection of *P. ramorum* in plants. Despite the lower sensitivity of LAMP compared to qPCR with the same marker, the reaction was successfully used to detect small amounts of the pathogen’s DNA in the sample [116]. The *YPT1* gene was used in order to compare it to the effectiveness of the nested PCR and LAMP assays to identify *P. melonis*. Consequently, both assays were c. 1 000 times more specific than PCR. The LAMP reaction was able to detect 10 fg of fungal DNA, thus it may be utilized in the early stages of infection [117]. Si Ammour’s team confirmed this thesis, as they detected *P. infestans* with LAMP 24 hours after the artificial inoculation of potato plants [118]. The efficiency of the marker in LAMP for *P. infestans* identification was reinforced by Khan’s team study. The team compared PCR with nested PCR, qPCR and LAMP with the application of the *YPT1* gene marker. LAMP was in fact the most sensitive reaction, being 10 times more sensitive than nested PCR and 100 times more sensitive than qPCR. What is more, the team detected the pathogen as soon as one hour after inoculation on the plant [119]. Taking under account the above mentioned results the detection limits of *Phytophthora* sp. was ranged from 1µg to 10 fg depending on selected method and tested species.

Whole genome sequencing of a few *Phytophthora* species has already been performed. In 2006, a draft of the genome sequences of *P. sojae* and *P. ramorum* were obtained. The genetic material of the fungi had a 9-fold coverage of the 95 Mb and a 7-fold coverage of the 65 Mb of *P. sojae* and *P. ramorum* genomes, respectively. The identification of a number of SNPs for both species was also achieved [120]. *P. infestans* whole-genome sequencing was also achieved with a 9-fold coverage assembly spanning 229 Mb of the pathogen’s genome [121]. Both of the abovementioned studies utilized a shot-gun approach, and the application of the Illumina platform was utilized for *P. rubi* and *P. fragariae*. The pathogen genetic material sequencing resulted in a 76-fold coverage of 5.88 Mb for *P. fragariae* and a 92-fold coverage of 6.96 Mb for *P. rubi* [122].

#### 4.2.3. *Botrytis Cinerea*

Rigotti’s team proposed specific primers for a RAPD assay in PCR for the detection of 13 strains of *Botrytis cinerea* in fields of symptomless strawberry plants. They proved that the presence of 0.2 pg of fungal DNA in the sample is enough for pathogen detection with this method [123]. The application of the SCAR assay was applied for the development of a specific marker for the detection of *B. cinerea, B. fabae,* and *B. fabiopsis*. The proposed primers were capable of distinguishing species from each other, as well as detecting 400 pg of *B. cinerea* in the reaction [124]. The markers mentioned above with the addition of the *ITS* region, glyceraldehyde-3-phosphate dehydrogenase gene (*G3PDH*), heat-shock protein 60 gene (*HSP60*) and DNA-dependent RNA polymerase subunit II gene (*RPB2*) markers were used for the identification of the strawberry pathogen. An analysis of the sequenced fragments showed that the disease was caused by *B. cinerea* [125]. In 2016, Kim’s team proposed *ITS* region amplification for the identification of pathogens causing grey mould on red raspberry. The reaction identified the pathogen as *B. cinerea*. However, for further investigation the sequencing of *G3PDH, HSP60*, and *RPB2* was performed. Those three protein-coding markers were also 100% identical with those of *B. cinerea*, confirming the identification of the fungus [126]. The *ITS* marker was also applied for the identification of the pathogen causing gray mould on economically important crops, including strawberry fruit. The analysis was also executed in connection with morphology identification and BIOLOG application. As a result, all of the methods confirmed that the pathogen was in fact *B. cinerea* [127]. Furthermore, the *ITS* region was also used for the identification of the strawberry postharvest pathogen in Pakistan. The method identified the fungus as *B. cinerea* [128]. Also, the amplification of the marker with PCR confirmed the presence of the fungus on *H. bracteatum* [129]. Another study published in 2018 included a phylogenic analysis of *B. cinerea* isolates obtained from strawberry cultivations. The sequences used in the study were 4 microsatellite markers and they contained enough phylogenic information for the analysis [35]. 

The application of *β-tubulin* and *actin* gene-specific markers were utilized for the quantification of *B. cinerea* on the *Arabidopsis thaliana* plant via a qPCR assay. Ten ng of fungal DNA was detectable for both of the markers [130]. Also, a different protein-coding gene marker—cutinase A gene was useful for the detection and quantification of *B. cinerea* from infected plants. The assay was capable of successfully detecting 16.7 ng of the genomic DNA of the pathogen [131]. Furthermore, Suarez’s team designed primers for *IGS*, the *β-tubulin* gene and the species-specific sequence-characterized amplified region (*SCAR*) genes of the fungus. Those regions were analyzed before and after the manifestation of the disease in order to detect and quantify the pathogen on strawberry plants. The application of the *IGS* and *SCAR* primers resulted in a high degree of specificity. What is more, the amplification of the IGS gene was the most sensitive method, detecting 20 fg of fungal DNA [132]. Furthermore, Reich’s team proved *IGS* primers to be useful in multiplex qPCR reactions for discrimination between *B. cinerea* and *Sclerotinia sclerotiorum* [133]. Multiplex qPCR for the simultaneous detection of the resistance of *B. cinerea* to benzimidazoles, dicarboximides, SDHIs, and SBIs was utilized in a recent study. The assay included the design of 4 specific pairs of primers for SNPs in genes responsible for the fungicide resistance, which are *β-tubulin*, succinate dehydrogenase iron-sulfur subunit (*SdhB*), putative osmosensor histidine kinase (*BcOS1*) and 3-ketoreductase (*erg27*) genes. The assay was capable to simultaneously detect all of the alleles when the concentration of genomic DNA was higher than 0.1 ng [134]. Primer sequences as well as the information of authors and targeted markers are described below in Table 4.

In 2010, the first LAMP reaction with *IGS* primers for *B. cinerea* detection was designed, and it resulted in a high level of efficiency. The assay was capable of detecting 65 pg of pathogen DNA in the sample, but for some of the reactions even an amount 10 times smaller was sufficient to detect the pathogen. The reaction also amplified only the closest related specimen, *Botrytis pelargonii*. What is more, detection was possible only 15 minutes after the start of the reaction [138]. Duan’s team proved the usefulness of the mitogen-activated protein kinase gene (*Bcos5*) which is an analysis designed to discriminate between *B. cinerea* on strawberry and tomato and 8 other plant pathogens in LAMP [139]. Also, LAMP assays for the detection of fungicide-resistant *B. cinerea* mutants have been developed [140,141,142]. Based on the presented above results concerning the detection limits of *B. cinerea* it was observed that depending on selected method and tested isolates the detection was within the limits between 17 ng and 20 fg.

The first genome sequencing of *B. cinerea* was obtained using Sanger technology, with the result of low coverage [143], which was a reason to search for more cost-effective and thorough methods. In 2012, Staats and van Kan employed Illumina technology to build an assembly with a size of *c.* 41 Mb, and a GC content of 42.5% [144]. Furthermore, a complete pathogen genome was accomplished with the final length of 43.5 Mb [145].

#### 4.2.4. *Colletotrichum acutatum*

*Colletotrichum acutatum* on berries has been identified with the application of a wide range of markers used in PCR reactions. In a study from 2009, cranberry fruit pathogens were detected with the application of the *ITS* region, which contained *ITS1*, 5.8S ribosomal RNA gene and *ITS2*. Also, an analysis of the partial sequence of the 28S ribosomal RNA gene—*LSU* was utilized. An analysis of the second marker resulted in an improved phylogeny for the species [146]. However, with strawberry pathogens the sequencing of the *ITS* region produced sufficient results for the differentiation of the fungus from *C. gleosporide* [52]. Additionally, the identification of strawberry pathogens in Belgium with the aforementioned marker was sufficient to distinguish between *C. acutatum, C. gloeosporioides* and *C. coccodes* [147]. Also, an analysis of a different marker—the *IGS* region, for 31 isolates of strawberry pathogens, as well the utilization of species-specific primers in PCR for *C. acutatum* was carried by Xie’s team. In agreement with the conclusions of Garrido and van Hemelrijck, the method was capable of identifying three species from the *Colletotrichum* genus, including *C. acutatum* [148]. A different approach, utilizing a restriction fragments length polymorphism (RFLP) protocol with glutamine synthetase (*GS*) introne marker also prevailed for the purpose of differentiating between both species [149]. The *ITS* region with the addition of the *β-tubulin* gene was also considered for the identification of the fungus isolated from different hosts. The *β-tubulin* based phylogeny tree had a higher resolution compared to that constructed with *ITS*, but both [150]. An extended number of markers were utilized for the identification of the causes of strawberry anthracnose in China. The application of primers directed for fragments of actin (*ACT*), *β-tubulin*, glyceraldehyde-3-phosphate dehydrogenase (*GPDH*), and chitin synthase (*CHS-1*) were satisfactory for distinguishing between *C. acutatum* and *C. gleosporide* [151]. The cytochrome b (*cytb*) gene was also utilized to reveal the fungicide resistance of the strawberry attacking pathogens [152].

The first application of *ITS* region and *β-tubulin* gene in a qPCR assay for the detection of *Colletotrichum acutatum* proved the specificity of the method. In the fungus genome the *β-tubulin* region exists only in one copy, in contrast with the multiple copies of the *ITS* region. Therefore, the method based on *ITS* marker was *c.* 66 times more sensitive and detected 50 fg of genomic DNA [153]. Furthermore, a duplex qPCR assay for the simultaneous detection of *C. godetiae* and *C. acutatum* was developed by Schena’s team. The method included the design of 2 pairs of specific primers, based on 2 markers: *β-tubulin* and *histone H3* genes. The presence of 10 pg of genomic DNA in the sample was enough to detect both species [154]. A summary of the most important information containing markers, primers sequences and authors of the assays is given in Table 5. 

The LAMP reaction was also used for the rapid identification of pathogens from different hosts, including strawberry and raspberry plants. Zhang’s team utilized previously designed primers for the ITS region and *β-tubulin 2* gene, with a greater specificity of the second marker. Nevertheless, the ITS marker was more sensitive, but it amplified the fragment for *C. acutatum, C. gloeosporioides* and *C. fragariae* [158].

The whole-genome sequence for *C. acutatum* has already been attained in 2016 by Han’s team. The team utilized NGS technology, and the final assembly was longer than 52 Mb, with a GC content of *c.* 51.5% [159].

## 5. Summary and Future Challenges

Soil-borne diseases are a serious threat to organic berry plantations, severely reducing crop yields. Until recently, the most effective way to prevent the spread of pathogenic fungi in the field was to immediately remove infected plants from the cultivation. Thus, fast and correct pathogen identification is essential for the eradication of the disease in time [12,21,160]. The accurate identification of pathogens can be problematic, as fungi attacking berries from the *Phytophthora* and *Verticillium* genera, as well *Botrytis cinerea* and *Colletotrichum acutatum* species cause similar symptoms on different plants and fruits. Identification based only on the morphology of the colonies is time-consuming and prone to misinterpretations, as it is based on human experience. These circumstances have led to the intense development of molecular techniques which allow for pathogen recognition and quantification [161]. Despite the fact that various molecular methods to detect fungi described in this review have already been established, they all have some disadvantages. These methods are only sensitive for a given region, also the majority of the assays are designed for pure strains. Those pure strains of fungi are more suitable for DNA isolation and recognition because the samples do not contain other closely related nor competitive microorganisms and their secretions, which often inhibit reactions. Therefore it is necessary to develop molecular methods that are more sensitive, specific and work under different soil and climatic conditions. Additionally, most of the pathogens causing agricultural losses cannot be grown in artificial cultures due to their specific environmental requirements, thus identification methods that do not necessitate the cultivation of pure cultures also have to be established.

## Figures and Tables

**Table 1:** Sequenced genomes of fungal pathogens from NCBI genome database.

Targeted Organism	Number of Genome Assemblies	Median Total Length (Mb)	Median Protein Count	Median GC%
*Verticillium dahliae*	11	33.2952	10393	55.6
*Verticillium alfalfae*	2	32.7521	10237	55.4
*Verticillium tricorpus*	2	35.5915	nd	57.4
*Verticillium nonalfalfae*	2	32.9671	9431	54.8
*Verticillium albo-atrum*	1	36.4685	nd	56.5
*Verticillium longisporum*	2	99.8546	20932	53.05
*Verticillium isaacii*	1	35.6909	nd	57.5
*Verticillium zaregamsianum*	1	37.1319	nd	57.5
*Verticillium klebahnii*	1	36.0824	nd	57.6
*Verticillium nubilum*	1	37.9116	nd	53.7
*Phytophthora infestans*	2	190.329	17797	36.9
*Phytophthora capsici*	7	56.0343	nd	49.9
*Phytophthora ramorum*	23	40.7668	nd	54
*Phytophthora nicotianae*	3	71.414	13910	50.2
*Phytophthora cactorum*	2	63.5331	24172	49.65
*Phytophthora rubi*	2	76.9186	nd	53.15
*Phytophthora fragariae*	2	76.4756	nd	53.2
*Phytophthora cinnamomi*	4	58.3834	nd	54
*Phytophthora parasitica*	9	54.2899	27003	49.6
*Phytophthora kernoviae*	11	38.1112	9990	50.3
*Phytophtora lateralis*	5	49.0253	nd	53.3
*Phytophthora palmivora*	1	107.773	24271	48.7
*Phytophthora sojae*	1	82.5976	26489	54.4
*Phytophthora litchii*	1	38.2009	nd	49.2
*Phytophthora colocasiae*	1	56.5926	nd	nd
*Phytophthora agathidicida*	2	37.2895	nd	52.6
*Phytophthora pluvialis*	2	53.178	nd	54.2
*Phytophthora multivora*	2	40.1961	nd	51.9
*Phytophthora pinifolia*	1	94.6173	nd	54.9
*Phytophthora cryptogea*	1	63.8393	nd	51.9
*Phytophthora cambivora*	1	230.616	nd	52.9
*Phytophthora plurivora*	1	40.4412	nd	51.7
*Phytophthora megakarya*	1	101.505	34804	48.7
*Phytophthora x alni*	1	236	nd	51.3
*Phytophthora pisi*	1	58.8567	nd	54.6
*Botrytis cinerea*	4	41.8726	13703	42.26
*Colletotrichum acutatum*	2	48.5246	nd	50.8

nd.—no data present.

**Table 2:** Selected primers designed for molecular analysis of *Verticillium* spp.

Targeted Organism (Number of Strains Analyzed)	Assay	Marker	Primers Sequences 5′-3′	Primers Authors	Primers Used in
*V. albo-atrum* (5) *V. tricorpus* (7)	multi-plex PCR	*ITS* ^1^	CCGGTCCATCAGTCTCTCTG CTGTTGCCGCTTCACTCG	[79]	[79]
*V. longisporum* (42)		*EF* ^2^	AAGTGGAGCCCCGTATCTTGAAT CAACTGGCAACAGGGCTTGAAT		
*V. isaacii* (14)			CGATGTCGCGATGACCTCG CGGCAGCCTCCTAAACATGG		
*V. klebahnii* (7)			ACATCCTGAGGCTGCTTGAGA CGGCAGCCTCCTAAACATGG		
*V. zaregam- sianum* (10)		*GPD* ^3^	GGTTTCCTCCCCTCACACG CCACCCTTGATGTGGGCGGA		
*V. longisporum* (42)			CCCCGGCCTTGGTCTGAT TGCCGGCATCGACCTTGG		
*V. alfalfae* (7)			TCATGCCCCCTTTGTTCATCGAT TGCCGGCATCGACCTTGG		
*V. albo-atrum* (5)		*ACT*	GGCCTCGATAGCATCGCC CTGGATGGAGACGTAGAAGGC		
*V. tricorpus* (5)			CGTGCTGTCTTCCGTAAGTTTG CTGGATGGAGACGTAGAAGGC		
*V. nonalfalfae* (9)		*TS* ^5^	CCTCGAAAAATCCACCAGCTCTA GTGGTTGAGATCCTCACGCTTC		
*V. nubilum* (4)			GGTCCCCCTCGTTCATGCAATC GTGGTTGAGATCCTCACGCTTC		
*V. dahliae* (10) *V. longisporum* (10)	PCR	*ITS* ^1^	GGA AGTAAAAGTCGTAACAAGG TCCTCCGCTTATTGATATGC	[91]	[19]
		*COX3* ^6^	TGATTTAGAGATST AATATCAGAAG CCGTGGAAACCTGTSCCAAAATA	[19]	
		NAD1 ^7^	ATGGCSAGTATGCAAAGAAGA GCATGTTC TGTCATAAASCCACTAAC	[92]	
*Verticillium* spp. (7)	qPCR	*ITS* ^1^	CTTGGTCATTTAGAGGAAGTAA AAAGTTTTAATGGTTCGCTAAGA	[80]	[80]
*V. tricorpus* (4)	qPCR	*ITS* ^1^	CCGGTGTTGGGGATCTACT GTAGGGGGTTTAGAGGCTG	[91]	[83]
*V. dahliae* (9) *+ V. longisporum*		*β-tubulin*	GGCCAGTGCGTAAGTTATTCT ATCTGGTTACCCTGTTCATCC	[84]	
*V. longisporum* (11)		*β-tubulin*	GCAAAACCCTACCGGGTTATG AGATATCCATCGGACTGTTCGTA		
*V. dahliae* (1) *+ V. longisporum* (1)	qPCR	*ITS* ^1^	CAGCGAAACGCGATATGTAG GGCTTGTAGGGGGTTTAGA	[93]	[93]
*V. dahliae* (44)	qPCR	*IGS* ^8^	CGTTTCCCGTTACTCTTCT GGATTTCGGCCCAGAAACT	[87]	[87]
*V. tricorpus* (13)		endochitinase	TAGTAGAATACTAGATARCTAG AGCCTAGGTCTTTATAGCTAG		
*V. dahliae*			CTCGGAGGTGCCATGTACTG ACTGCCTGGCCCAGGTTC		
*V. dahliae*		*β*-tubulin	GCGACCTTAACCACCTCGTT CGCGGCTGGTCAGAGGA		
		*G3PD* ^9^	CACGGCGTCTTCAAGGGT CAGTGGACTCGACGACGTAC		
		*VTP1* ^10^	GCGGTGGCTGGTTCCTATCAAC CAACGACTTCGCCATCTGGAAG		
*V. albo-atrum* group 1 (isolation from soil)	qPCR	actin	GCCCTCTTCCAGCCCTCCGTTCTC TCGGCGTGGTTTTGTGGTGAG	[87]	[94]
*V. albo-atrum* group 2 (isolation from soil)	qPCR	*IGS* ^8^	CGTGTTTAGTGTATTTCACCCTTG TCGCAGAGTAGTACGATTTCTC	[94]	
*V. longisporum* (isolation from soil)			CGAGGAGTGAAAAGAAAACGGTTA CGCGCCGAGGCTAGTCAC		
*V. dahliae* (5)	qPCR	not explained in the study	TCCTAGGCAGGCGAGCAG TAGGGCTGTCTGTCGGTGA	[90]	[90]
*V. albo-atrum* (4)			TTTCACGACCGATGAAAGCG CACATCGGCGAGGATCTGTC		
*V. tricopus* (4)			CACCCTCGGGCACACCAATA TCCGTGGAGGTTGAGCGCTAT		
*V. longisporum* (4)			CGAGGAGTGAAAAGAAAACGGTTA CGCGCCGAGGCTAGTCAC		

^1^ internal transcribed spacer, ^2^ elongation factor 1-α, ^3^ glyceraldehyde-3-phosphate dehydrogenase, ^4^ actine, ^5^ tryptophan synthase, ^6^ cytochrome oxidase subunit III, ^7^ NADH dehydrogenase subunit 1, ^8^ intergenic spacer of genomic DNA, ^9^ glyceraldehyde-3-phosphate dehydrogenase, ^10^ extracellular trypsin protease.

**Table 3:** Selected primers designed for molecular analysis of *Phytophthora* spp.

Targeted Organism (No. of Strains Analyzed)	Assay	Marker	Primers Sequences 5′-3′	Primer Authors	Primers Used in
*Phytophthora* spp. (15 in [101]; 14 in [102])	PCR	*ITS* ^1^	GGAAGTAAAAGTCGTAACAAGG TCCTCCGCTTATTGATATGC	[91]	[101,102]
*Phytophthora* spp. (51)	PCR	*COX1* ^2^	GCGTGGACCTGGAATGACTA AGGTTGTATTAAAGTTTCGATCG	[114]	[114]
		*COX2* ^3^	AAAAGAGAAGGTGTTTTTTATGGA GCAAAAGCACTAAAAATTAAATATAA		
*P. ramorum*	nested PCR	Spacer sequences between the *COX 2* and *COX1* gene	GTATTTAAAATCATAGGTGTAATTTG TGGTTTTTTTAATTTATATTATCAATG		
*P. nemorosa*			AATAAAATTAATTTTAATATATAATTAG TATGTTTAATATCTGTAAATAATAG		
*P. pseudo- syringae*			CAGTTTCATTAGAAGATTATTTAC AAAATTGTTTGATTTTATTAAGTATC		
*Phytophthora* spp. (82)	PCR	60S ribosomal protein L10	GCTAAGTGTTACCGTTTCCAG ACTTCTTGGAGCCCAGCAC	[106]	[106]
		*β-tubulin*	GCCAAGTTCTGGGARGTSAT GCCAAGTTCTGGGARGTSAT		
		Enolase	CTTTGACTCGCGTGGCAAC CCTCCTCAATACGMAGAAGC		
		Heat shock protein 90	GCTGGACACGGACAAGAACC CGTGTCGTACAGCAGCCAGA		
		*tigA* gene fusion	TTCGTGGGCGGYAACTGG TCGTGGGCGGYAAYTGGAA GCCTACATCACGGAGCARA TCGCYATCAACGGMTTCGG CCGAAKCCGTTGATRGCGA GCCCCACTCRTTGTCRTACCAC		
		EF ^4^	GGTCACCTGATCTACAAGTGC CCTTCTTGTTCACCGACTTG		
*P. infestans* (1)	qPCR	GC-rich nuclear satellite DNA with unknown function	GCCAT CAAGACGTGCGAGA GCAGGGATTCGGGCATA	[108]	[108]
*P. citricola* (1)		*ITS* ^1^	TCAACCCTTTTAGTTGGGGGTC TTTAAAACAAAAAGCTACTAGCCCAGAC		
*Phytophthora* spp. (71)	qPCR	*ITS* ^1^	TGCGGAAAGGATCATTACCACACC GCGAGCCTAGACATCCACTG	[109]	[109]
*P. colocasiae* (49)	qPCR	*YPT1* ^5^	GGTGTGGACTTTGTGAGTTTCAG AAGGGAGTTGGCACAACCATT	[111]	[111]
		*TRP1* ^6^	AGCGCCTTAACGCTCCCT GAGCCCTTGAACCACTTGGG		
		*GPA1* ^7^	TTGGTGGCGTGTAGTCTGTG AGCTTCCGGTTGATGGTAGC		
*Phytophthora* spp. (15)	qPCR	*YPT1* ^4^	ATGAACCCCGAATAGTRCGTGC TGTTSACGTTCTCRCAGGCG	[115]	[115]
		*TRP1* ^6^	GAGGAGATCGCGGCGCAGCG GCGCACATRCCGAGVTTGTG		
		*GPA1* ^7^	GGACTCTGTGCGTCCCAGATG ATAATTGGTGTGCAGTGCCGC		
*P. nicotianae* (7)	qPCR	*ITS* ^1^	CCTATCAAAAAAAAGGCGAACG TACACGGAAGGAAGAAAGTCAAG	[112]	[112]
*P. cactorum* (7)		*YPT1* ^5^	CATGGCATTATCGTGGTGTA GCTCTTTTCCGTCGGC		

^1^ internal transcribed spacer, ^2^ cytochrome oxidase subunit I, ^3^ cytochrome oxidase subunit II, ^4^ Elongation factor 1-α, ^5^ RAS-related protein, ^6^ phosphoribosylanthranilate isomerase, ^7^ G protein α-subunit.

**Table 4:** Selected primers designed for molecular analysis of *Botrytis* spp.

Targeted species (No. of Strains Analyzed)	Assay	Marker	Primers Sequences 5′-3′	Primer Authors	Primers Used in
*Botrytis* spp. (1)	PCR	*ITS* ^1^	GGAAGTAAAAGTCGTAACAAGG TCCTCCGCTTATTGATATGC	[91]	[125]
*B. cinerea* (13)	PCR	*ITS* ^1^	ACCCGCACCTAATTCGTCAAC GGGTCTTCGATACGGGAGAA	[123]	[123]
*B. cinerea* (29)	PCR	RAPD ^2^ marker	CAGGAAACACTTTTGGGGATA GAGGGACAAGAAAATCGACTAA	[124]	[124]
*B. fabae* (8)		*NEP1* ^3^	TCACGGTTTCTTGTCCATCC TCGGGCGTTGTACTCTTCAT		
*B. fabiopsis* (8)		RAPD^2^ marker	TCCTTTCTATCCTCGCTGCC CTGGTGGTTTGTAAAGCTGC		
*Botrytis* spp. (52)	PCR	*RPB2* ^4^	GATGATCGTGATCATTTCGG CCCATAGCTTGCTTACCCAT	[135]	[135]
		*G3PDH* ^5^	ATTGACATCGTCGCTGTCAACGA ACCCCACTCGTTGTCGTACCA		
		*HSP60* ^6^	CAACAATTGAGATTTGCCCACAAG GATGGATCCAGTGGTACCGAGCAT		
*B. cinerea* (39 in [49]; 273 in [35])	PCR	microsatellite marker	ACCCGCACCTAATTCGTCAAC GGGTCTTCGATACGGGAGAA	[49]	[35]
*B. cinerea* (117 in [136])	PCR	microsatellite marker	AAGCCCTTCGATGTCTTGGA ACGGATTCCGAACTAAGTAA	[136]	[35]
*B. cinerea* (75 in [137])	PCR	microsatellite marker	AGGGAGGGTATGAGTGTGTA TTGAGGAGGTGGAAGTTGTA	[137]	[35]
		microsatellite marker	CATACACGTATTTCTTCCAA TTTACGAGTGTTTTTGTTAG		
		microsatellite marker	GGATGAATCAGTTGTTTGTG CACCTAGGTATTTCCTGGTA		
		microsatellite marker	CATCTTCTGGGAACGCACAT ATCCACCCCCAAACGATTGT		
		microsatellite marker	CGTTTTCCAGCATTTCAAGT CATCTCATATTCGTTCCTCA		
		microsatellite marker	ACTAGATTCGAGATTCAGTT AAGGTGGTATGAGCGGTTTA		
		microsatellite marker	CCAGTTTCGAGGAGGTCCAC GCCTTAGCGGATGTGAGGTA		
		microsatellite marker	CTCGTCATAACCACGCAGAT GCAAGGTCTCGATGTCGATC		
		microsatellite marker	TCCTCTTCCCTCCCATCAAC GGATCTGCGTGGTTATGACG		
*B. cinerea* (1)	qPCR	*β-tubulin*	CCGTCATGTCCGGTGTTACCAC CGACCGTTACGGAAATCGGAAG	[130]	[130]
		*actin*	TGGAGATGAAGCGCAATCCAA AAGCGTAAAGGGAGAGGACGG		
*B. cinerea* (1)	qPCR	*cutinase A*	AGCCTTATGTCCCTTCCCTTGCG GAAGAGAAATGGAAAATGGTGAG	[131]	[131]
*B. cinerea* (24)	qPCR	*β-tubulin*	GTTACTTGACATGCTCTGCCATT CACGGCTACAGAAAGTTAGTTTCTACAA	[132]	[132]
		*IGS* ^7^	FGCTGTAATTTCAATGTGCAGAATCC GGAGCAACAATTAATCGCATTTC		
		*SCAR*^8^ marker	TTCGTGATTATCACCTGGGTTG GCTCCTAGAACGTACGACCACA	[123]	
*B. cinerea* (11)	multi-plex qPCR	*β-tubulin*	GTCGTCCCATCGCCAAAGGT ACGGTGACAGCACGGAAAGA	[134]	[134]
		*SdhB* ^9^	ACACCGACCCAGCACCAGA TTAGCAATAACCGCCCAAA		
		*BcOS1* ^10^	AGGTCACCCGCGTAGCAAGA TGCTTGATTTCACCCTTACA		
		*erg27* ^11^	GCGTGGAGAACTCTAAATCGG AGTGTAAGGCTTGATGGTATGC		

^1^ internal transcribed spacer, ^2^ random amplification of polymorphic DNA, ^3^ necrosis-and ethylene-inducing protein 1, ^4^ DNA-dependent RNA polymerase subunit II gene, ^5^ glyceraldehyde-3-phosphate dehydrogenase gene, ^6^ heat-shock protein 60 gene, ^7^ intergenic spacer of genomic DNA, ^8^ sequence characterized amplified region, ^9^ succinate dehydrogenase iron–sulfur subunit, ^10^ putative osmosensor histidine kinase, ^11^ 3-ketoreductase.

**Table 5:** Selected primers designed for molecular analysis of *Colletotrichum* spp.

Targeted species (No. of Strains Analyzed)	Assay	Marker	Primers Sequences 5′-3′	Primer Authors	Primers Used in
*C. acutatum* (16 in [146])	PCR	*ITS* ^1^	GGAAGTAAAAGTCGTAACAAGG TCCTCCGCTTATTGATATGC	[91]	[146]
*C. acutatum* (16 in [146])	PCR	*LSU* ^2^	ATCCTGAGGGAAACTTC AGATCTTGGTGGTAGTA	[155]	[146]
*Colletotrichum* spp. (29)	PCR	*ITS* ^1^	AACCCTTTGTGAACRTACCTA TTACTACGCAAAGGAGGCT	[156]	[156]
*Colletotrichum* spp. (100 in [151]	PCR	*GPDH* ^3^	TCCCATCAAGGTCGGCATCA ACCTTGCCGACAGCCTTGG	[157]	[151]
		*CHS-1* ^4^	GATGCCTGGAAGAAGATTGTCGT GTCTCGCCAGTAGCGGACTTGAC		
		*CAL ^5^*	GAATTCAAGGAGGCCTTCTC CTTCTGCATCATGAGCTGGAC		
*C. acutatum* (181)	PCR	*Cytb ^6^*	GAAGAGGTATGTACTACGGTTCATATAG TAGCAGCTGGAGTTTGCATAG	[152]	[152]
*C. acutatum* (23 in [70])	qPCR	*ITS* ^1^	CGGAGGAAACCAAACTCTATTTACA CCAGAACCAAGAGATCCGTTG	[91]	[70]
*C. acutatum* (6)	qPCR	*ITS* ^1^	GGATCATTACTGAGTTACCGC GCCCACGAGAGGCTTC	[153]	[153]
		*β-tubulin*	CGTCTACTTCAACGAAGTTTGTTATCC GAGGCCTGGTTGGGTGAG		
*C. acutatum* (15)	qPCR	*histone H3*	TCCAGCGTCTGGTAAGTTGAGAA AGAAGTGTTAGCCGATGCGATT	[154]	[154]

^1^ internal transcribed spacer, ^2^ partial sequence of the 28S ribosomal RNA gene, ^3^ glyceraldehyde-3-phosphate dehydrogenase, ^3^ chitin synthase, ^4^ calmoduline, ^5^ cytochrome b.
